# Docosahexaenoic Acid Reduces the Incidence of Early Afterdepolarizations Caused by Oxidative Stress in Rabbit Ventricular Myocytes

**DOI:** 10.3389/fphys.2012.00252

**Published:** 2012-07-09

**Authors:** Zhenghang Zhao, Hairuo Wen, Nadezhda Fefelova, Charelle Allen, Nancy Guillaume, Dandan Xiao, Chen Huang, Weijin Zang, Judith K. Gwathmey, Lai-Hua Xie

**Affiliations:** ^1^Department of Pharmacology, School of Medicine, Xi’an Jiaotong UniversityXi’an China; ^2^Department of Cell Biology and Molecular Medicine, UMDNJ-New Jersey Medical SchoolNewark, NJ, USA; ^3^Gwathmey Inc.Cambridge, MA, USA; ^4^School of Optometry, Massachusetts College of Pharmacy and Health SciencesWorcester, MA, USA

**Keywords:** docosahexaenoic acid, H_2_O_2_, early afterdepolarizations, reactive oxygen species, L-type calcium channel, sodium channel

## Abstract

Accumulating evidence has suggested that ω3-polyunsaturated fatty acids (ω3-PUFAs) may have beneficial effects in the prevention/treatment of cardiovascular diseases, while controversies still remain regarding their anti-arrhythmic potential. It is not clear yet whether ω-3-PUFAs can suppress early afterdepolarizations (EADs) induced by oxidative stress. In the present study, we recorded action potentials using the patch-clamp technique in ventricular myocytes isolated from rabbit hearts. The treatment of myocytes with H_2_O_2_ (200 μM) prolonged AP durations and induced EADs, which were significantly suppressed by docosahexaenoic acid (DHA, 10 or 25 μM; *n* = 8). To reveal the ionic mechanisms, we examined the effects of DHA on L-type calcium currents (*I*_Ca.L_), late sodium (*I*_Na_), and transient outward potassium currents (*I*_to_) in ventricular myocytes pretreated with H_2_O_2_. H_2_O_2_ (200 μM) increased *I*_Ca.L_ by 46.4% from control (−8.4 ± 1.4 pA/pF) to a peak level (−12.3 ± 1.8 pA/pF, *n* = 6, *p* < 0.01) after 6 min of H_2_O_2_ perfusion. H_2_O_2_-enhanced *I*_Ca.L_ was significantly reduced by DHA (25 μM; −7.1 ± 0.9 pA/pF, *n* = 6, *p* < 0.01). Similarly, H_2_O_2_-increased the late *I*_Na_ (−3.2 ± 0.3 pC) from control level (−0.7 ± 0.1 pC). DHA (25 μM) completely reversed the H_2_O_2_-induced increase in late *I*_Na_ (to −0.8 ± 0.2 pC, *n* = 5). H_2_O_2_ also increased the peak amplitude of and the steady state *I*_to_ from 8.9 ± 1.0 and 2.16 ± 0.25 pA/pF to 12.8 ± 1.21 and 3.13 ± 0.47 pA/pF respectively (*n* = 6, *p* < 0.01, however, treatment with DHA (25 μM) did not produce significant effects on current amplitudes and dynamics of *I*_to_ altered by H_2_O_2_. In addition, DHA (25 μM) did not affect the increase of intracellular reactive oxygen species (ROS) levels induced by H_2_O_2_ in rabbit ventricular myocytes. These findings demonstrate that DHA suppresses exogenous H_2_O_2_-induced EADs mainly by modulating membrane ion channel functions, while its direct effect on ROS may play a less prominent role.

## Introduction

Extensive studies on the potential effects of fish oil omega-3 poly unsaturated fatty acids (ω-3 PUFA) on cardiac rhythm have provided controversial results (von Schacky, 2008). While some interventional studies reported either no effect or even promotion of arrhythmias in some subgroups of patients with heart disease (Raitt et al., 2005; Coronel et al., 2007; Den Ruijter et al., 2007; Cheng and Santoni, 2008), other studies have reported beneficial effects of ω-3-PUFAs on cardiac rhythm resulting in a reduction in the incidence of sudden cardiac death or mortality (London et al., 2007; Cheng and Santoni, 2008; Nodari et al., 2009). It seems that fish oil fatty acids may exert either pro- or anti-arrhythmic effects, probably depending on different underlying mechanisms for the arrhythmias. Recent studies have also shown ω-3-PUFAs suppress afterdepolarizations and triggered activities induced by K channel blockers or by β-adrenergic stimulation in failing hearts (Den Ruijter et al., 2006, 2008; Berecki et al., 2007; Smith et al., 2009). However, it is unclear whether ω-3-PUFAs have protective effects on arrhythmias induced by oxidative stress. Reactive oxygen species (ROS) have recently been implicated in the pathogenesis of cardiac arrhythmia during ischemic-reperfusion, aging, and heart failure. Oxidative stress caused by exogenous H_2_O_2_ induces early afterdepolarizations (EADs) and delayed afterdepolarizations (DADs) that may in turn trigger lethal arrhythmias. These afterdepolarizations are a result of a net increase in inward current, which is induced by activation of late sodium current (*I*_Na_) and the l-type calcium current (*I*_CaL_) via oxidized Ca^2+^/Calmodulin-Dependent Protein Kinase II (CaMKII; Ward and Giles, 1997; Xie et al., 2009; Zhao et al., 2011). Our most recent study suggested that the transient outward potassium current (*I*_to_) may also facilitate EAD generation by H_2_O_2_ (Zhao et al., 2012b)_._ In the present study, we aim to assess the effects of docosahexaenoic acid (DHA, one of ω-3-PUFAs) on exogenous H_2_O_2_-induced EADs, and to further reveal potential underlying ionic mechanisms.

## Materials and Methods

This investigation conforms to the Guide for the Care and Use of Laboratory Animals, published by the National Institutes of Health (NIH Publication No. 85–23, Revised 1996). All animal experimental procedures were reviewed and approved by the Institutional Animal Care and Use Committee at the University of Medicine and Dentistry of New Jersey-New Jersey Medical School and by the Ethical Committee of Xi’an Jiaotong University. All experiments were performed at 35–37°C.

### Cell isolation

Ventricular myocytes were enzymatically isolated from the hearts of New Zealand white rabbits (male, 1.8–2.5 kg) as described previously (Xie et al., 2009). Briefly, after rabbits were anesthetized with intravenous pentobarbital hearts were removed and perfused retrogradely at 37°C in Langendorff fashion with nominally Ca^2+^-free Tyrode’s solution containing 1.4 mg/mL collagenase (Type II; Worthington) and 0.1 mg/ml protease (type XIV, Sigma) for 25–30 min. The hearts were removed from the perfusion apparatus after washing out the enzyme solution, the left ventricles were gently teased apart with forceps in a Petri dish and the myocytes were filtered through a nylon mesh. The Ca^2+^ concentration was gradually increased to 1.8 mM, and the cells were stored at room temperature and used within 8 h.

### Electrophysiological recording

Myocytes were current-or voltage-clamped using the perforated whole-cell patch-clamp technique (240 μg/ml amphotericin B; Rae et al., 1991) for recordings of action potential, or *I*_Ca.L_, *I*_to_, and late *I*_Na_. Voltage or current signals were measured with a MultiClamp 700A patch-clamp amplifier controlled by a personal computer using a Digidata 1322 acquisition board driven by pCLAMP 10 software (Molecular Devices, Sunnyvale, CA, USA).

To record action potentials (APs), patch pipettes (resistance 2–4 MΩ) were filled with an internal solution containing (in mM): 110 K-aspartate, 30 KCl, 5 NaCl, 10 HEPES, 0.1 EGTA, 5 MgATP, 5 Na_2_-phosphocreatine, 0.05 cAMP, pH was adjusted to 7.2 with KOH. The cells were superfused with Tyrode’s solution containing (in mM): 136 NaCl, 4.0 KCl, 0.33 Na_2_PO_4_, 1.8 CaCl_2_, 1 MgCl_2_, 10 glucose, and 10 HEPES, pH was adjusted to 7.4 with NaOH. APs were elicited with 2 ms, 2 to 4 nA square pulses at a pacing cycle length (PCL) of 6 s.

To record the *I*_Ca,L_, patch pipettes (2–4 MΩ) were filled with an internal solution containing (in mM): 110 Cs-Aspartate, 30 CsCl, 5 NaCl, 10 HEPES, 0.1 EGTA, 5 MgATP, 5 Na_2_-phosphocreatine, 0.05 cAMP, pH 7.2 with CsOH, and the cells were perfused with a modified Tyrode’s solution, in which KCl was replaced with CsCl. The myocytes were stimulated at a PCL of 6 s with a double-pulse protocol. Following a 100-ms prepulse to −40 mV from the holding potential of −80 mV (to inactivate Na^+^ current and T-type Ca^2+^ current), *I*_Ca.L_ was elicited by a subsequent test depolarization step to 0 mV for 300 ms.

Late *I*_Na_ was measured as described previously (Song et al., 2006). Glass pipettes (1–2 MΩ) were filled with an internal solution containing (in mM): 110 Cs-Aspartate, 30 CsCl, 10 HEPES, 0.5 EGTA, 0.2 Na_3_-GTP, 5 Na_2_-phosphocreatine-, 5 MgATP, pH 7.2 was adjusted with CsOH. Myocytes were bathed with a modified Tyrode’s solution in which KCl was replaced with CsCl. Nifedipine (30 μM) was added to the bath solution to block calcium channels. Late *I*_Na_ was elicited by 300 ms voltage-clamp pulses from −90 to −30 mV at a PCL of 6 s from a holding potential of −80 mV.

To record *I*_to_, the pipette and superfusion solutions were the same as those for AP recording. Tetrodotoxin (TTX, 10 μM) and CdCl_2_ (0.5 mM) were added into the Tyrode’s solution to inhibit *I*_Na_ and *I*_Ca,L_. *I*_to_ was evoked by 400 ms depolarizing pulses to test potentials between −40 and +50 mV (0.1 Hz). The holding potential was set at −80 mV and a 100 ms prepulse was applied to −60 mV to inactivate the *I*_Na_. *I*_to_ recovery from inactivation was investigated using a conventional two-pulse protocol: an inactivating pulse depolarizing to +50 mV for 400 ms (P1) followed by a variable recovery interval and subsequent +50 mV test pulse (P2). The inactivation of *I*_to_ and recovery from inactivation were best fit with a double exponential equation. All electrophysiological data were normalized as current densities by dividing measured current amplitude by whole-cell capacitance.

All chemicals were purchased from Sigma-Aldrich unless indicated. Because DHA is very sensitive to oxidation, DHA (Sigma-Aldrich) was dissolved in 100% ethanol under N_2_ and kept at −20°C in the dark. Immediately before use, the DHA stock solution was diluted in the bath solution to reach the final concentrations needed. The maximum final concentration (0.1%) of ethanol had no effect on membrane currents.

### Measurement of intracellular ROS

The myocytes were incubated with 5 μM C-DCDHF-DA-AM (Invitrogen) for 30 min. C-DCDHF- DA is oxidized by ROS to dichlorofluorescein (DCF). ROS fluorescence (emission: ∼530 nm) was measured by a 200 ms-exposure (excitation: ∼480 nm) every 30 s using the Andor Ixon charge-coupled device camera. Recordings were started after a stable baseline was achieved.

### Statistical analysis

Data are presented as mean ± SEM. Differences were tested for statistical significance by using paired or unpaired Student’s *t* tests, with *p *< 0.05 considered significant.

## Results

### DHA suppresses the EADs induced by H_2_O_2_

**Figure 1:**
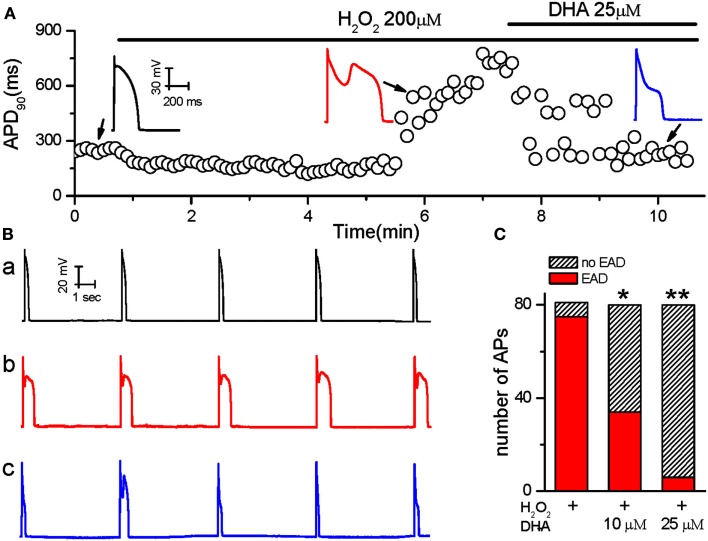
**The inhibitory effects of DHA on Early afterdepolarizations (EADs) induced by H_2_O_2_**. **(A)** Values of consecutive APD_90_ are plotted over time. The ventricular myocyte was treated with H_2_O_2_ and DHA as indicated by the horizontal bars above the plot. Three representative AP recordings under different conditions are shown in the insets. **(B)** Five consecutive AP recordings from a cell exposed to control perfusate **(a)**, 200 μM H_2_O_2_ **(b)** and 200 μM H_2_O_2_ + 25 μM DHA **(c)**. **(C)** Summarized bar graph showing dose-dependent inhibitory effects of DHA on the incidence of EADs induced by H_2_O_2_ (*n* = 8 cells). *p < 0.05, **p < 0.01; Fisher’s Exact Test vs. H_2_O_2_.

Action potentials were recorded from single ventricular myocytes isolated from rabbit hearts using the perforated whole-cell patch-clamp technique under current-clamp mode. In order to reliably induce EADs, the cells were paced at a PCL of 6 s based on our previous studies (Sato et al., 2009; Xie et al., 2009; Zhao et al., 2012a). The average APD_90_ of rabbit ventricular myocytes is 266 ± 23 ms (*n* = 8) at base line. After APD and morphology reached steady state, the cells were perfused with 200 μM H_2_O_2_ until EADs consistently appeared. Consecutively, DHA at either 10 or 25 μM was added in the presence of H_2_O_2_. The sudden and dramatic increase in APD_90_ in Figure 1A indicates the incidence of EADs. As shown in Figures 1A,B, EADs were consistently induced by H_2_O_2_ at 5 min after perfusion. DHA (25 μM) shortened the APD prolongation from 894 ± 78 ms to 278 ± 52 ms, and significantly suppressed the frequency of EADs induced by H_2_O_2_. The incidence of EADs was assessed by counting the number of EADs within 10 APs (from eight cells) in control, after H_2_O_2_ (200 μM) and H_2_O_2_ (200 μM) + DHA (at 10 or 25 μM). The incidence of EAD was suppressed in all tested cells (*n* = 8), five of which showed complete abolishment of EADs after 3–5 min of treatment with 25 μM DHA. As summarized in Figure 1C, the incidence of H_2_O_2_-induced EADs were significantly reduced by direct perfusion of DHA at both 10 and 25 μM, in a dose-dependent manner (*p* < 0.05 and *p* < 0.01, respectively, Fisher’s exact test).

### Inhibitory effect of DHA on *I*_CaL_ enhanced by H_2_O_2_

**Figure 2:**
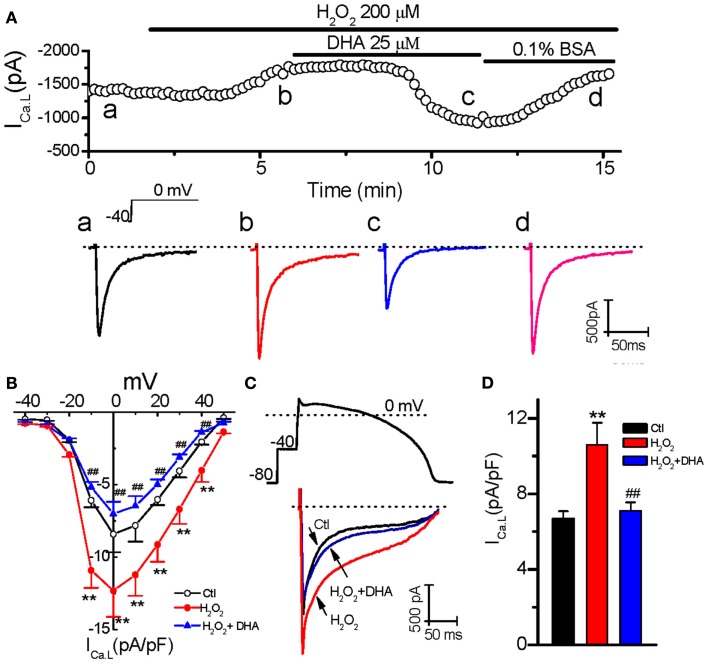
**Inhibitory effects of DHA on *I*_Ca.L_ enhanced by H_2_O_2_**. **(A)** Time course of peak *I*_Ca,L_ in a myocyte treated with 200 μM H_2_O_2_ in the absence and presence of 25 μM DHA, and 0.1% bovine serum albumin (BSA). Representative traces of *I*_Ca,L_ corresponding to points a–d are shown under the plot. **(B)** The current-voltage relations for peak *I*_Ca,L_ from six cells treated with 200 μM H_2_O_2_ in the absence and presence of 25 μM DHA. Test potentials ranged from −40 to +50 mV in 10 mV steps. **(C)** An AP-clamp waveform (above) and superimposed current traces showing *I*_Ca,L_ under control (Ctl), in the presence of 200 μM H_2_O_2_, and DHA(25 μM) + H_2_O_2_ are shown respectively. **(D)** The late phase currents measured at 30-150 ms after the upstroke in **(C)** were summarized showing an inhibitory effect of DHA on the enhancement of *I*_Ca,L_ by H_2_O_2_ (*n* = 6). **p < 0.01 vs. control; ## *p* < 0.01 vs. H_2_O_2_ group.

Our previous studies have shown that reactivation of *I*_Ca.L_ plays a key role in H_2_O_2_-induced EAD in rabbit ventricular myocytes (Xie et al., 2009; Song et al., 2010). Therefore, we first assessed the potential involvement of *I*_Ca.L_ in the inhibitory effect of DHA on H_2_O_2_-induced EADs. *I*_Ca.L_ was recorded in rabbit ventricular myocytes using the perforated whole-cell patch-clamp technique under voltage-clamp mode. As shown in Figure 2A, H_2_O_2_ (200 μM) gradually increased the amplitude of *I*_Ca.L_ at both peak and late phases (at ∼ 250 ms), which reached the steady state at 5–7 min, consistent with the time course for EAD induction as shown in Figure 1. The I-V relations for the peak current (Figure 2B) showed that the *I*_Ca.L_ amplitude was pronouncedly increased at testing potentials −10 to +40 mV. For example a 46.4% enhancement was caused at 0 mV, i.e., from −8.4 ± 1.4 to −12.3 ± 1.8 pA/pF (*n* = 6, *p* < 0.01). DHA (25 μM) significantly suppressed/reversed the elevation of the *I*_Ca,L_ amplitude (e.g., to −7.1 ± 0.9 pA/pF at 0 mV; *n* = 6, *p* < 0.01 compared to H_2_O_2_-induced effect). In order to test the DHA effect on *I*_Ca.L_ under normal membrane potential conditions, we also performed AP-clamp experiments. As shown in Figures 2C,D, DHA markedly decreased both the peak and the late phase of *I*_Ca.L_, which were enhanced by H_2_O_2_, under AP-clamp conditions.

### Inhibitory effect of DHA on late sodium current increased by H_2_O_2_

**Figure 3:**
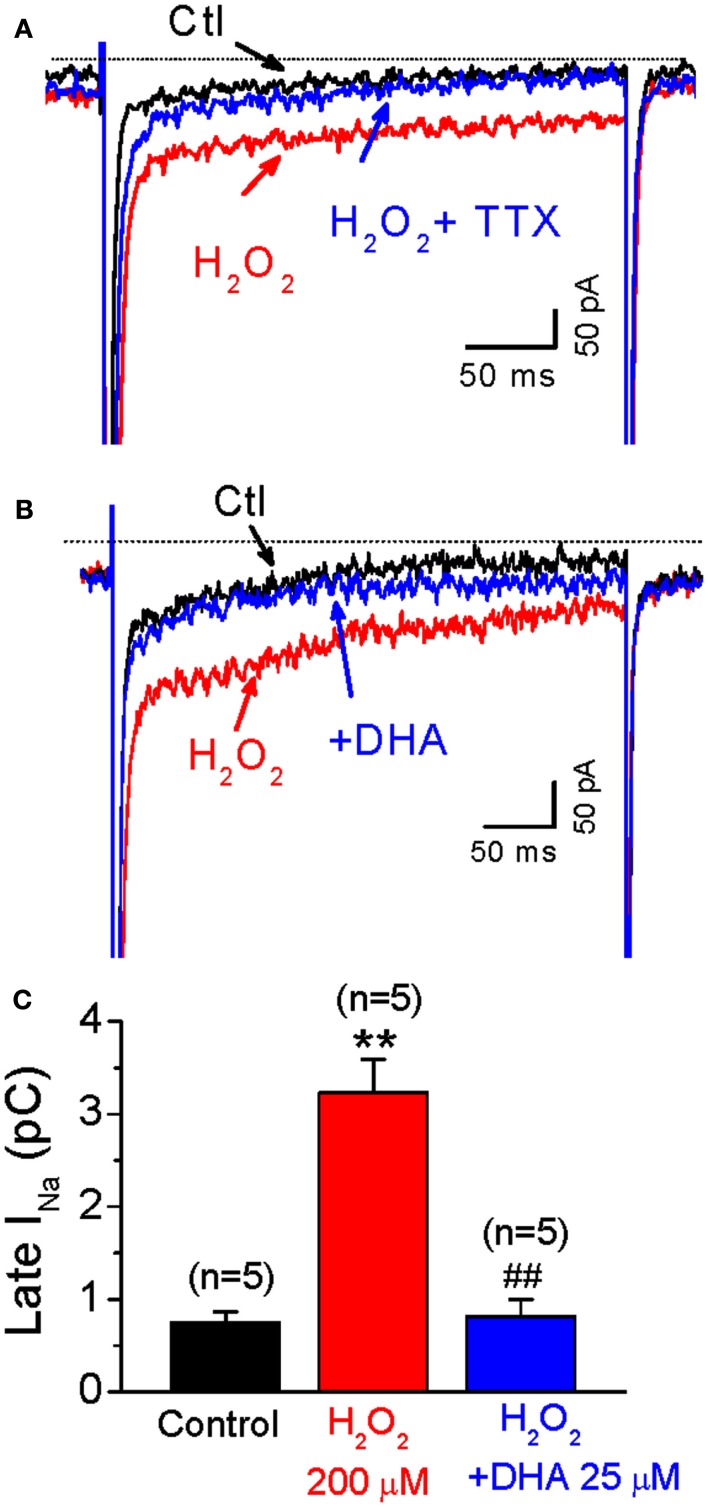
**Inhibitory effects of DHA on late *I*_Na_ enhanced by H_2_O_2_**. **(A)** Representative *I*_Na_ traces under control condition (Ctl), in the presence of 200 μM H_2_O, and H_2_O_2_ + TTX (10 μM), respectively. **(B)** Representative *I*_Na_ traces under control condition (Ctl), in the presence of 200 μM H_2_O, and H_2_O_2_ + DHA (25 μM), respectively. **(C)** A bar graph summarizing 200 μM H_2_O_2_-induced increase of late *I*_Na_, which is significantly suppressed by 25 μM DHA. **p < 0.01 vs. control; ## *p* < 0.01 vs. H_2_O_2_ group.

Since the activation of late *I*_Na_ also contributes to EAD generation induced by H_2_O_2_ (Ward and Giles, 1997; Xie et al., 2009), we next evaluated the effect of DHA on H_2_O_2_-enhanced late *I*_Na._ Late *I*_Na_ was elicited by 300 ms voltage-clamp pulses from −90 to −30 mV at a PCL of 6 s. The magnitude of late *I*_Na_ was evaluated by integration of the area (nA × ms = pC) of the current over the last 50 ms of the −30 mV depolarizing pulse, using the integration (area) feature of the pCLAMP program. As shown in Figure 3, the late current component was significantly enhanced by H_2_O_2_ (200 μM) from −0.7 ± 0.1 pC to −3.2 ± 0.3 pC (*n* = 5, *p* < 0.01) at 4–6 min after perfusion, when it reaches steady state level. This elevation was completely suppressed by Tetrodotoxin (TTX, 10 μM), a selective *I*_Na_ inhibitor, confirming this late sustained inward current is due to late *I*_Na_, although we cannot exclude minor contaminations on the baseline current from other currents such as Na-Ca exchange current (*I*_NCX_), *I*_Ca,L_ or leaky current. H_2_O_2_-increased late *I*_Na_ was effectively attenuated by 25 μM DHA (to −0.8 ± 0.2 pC at 2–4 min after DHA application, *n* = 5, *p* < 0.01).

### Effect of DHA on *I*_to_ increased by H_2_O_2_

**Figure 4:**
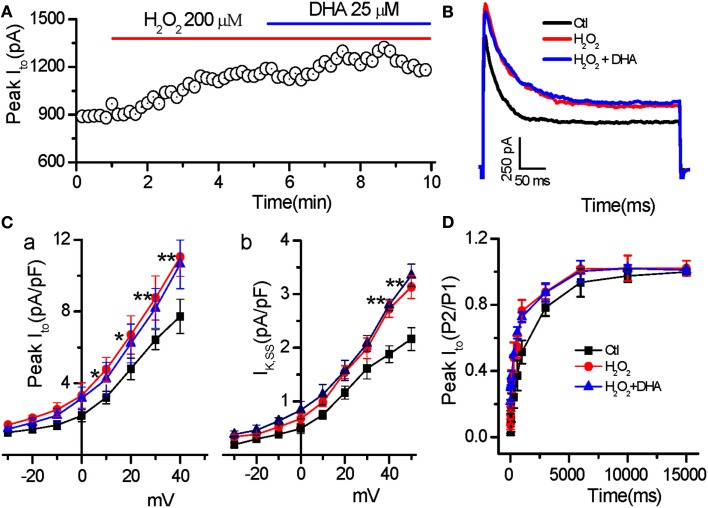
**Less effect of DHA on *I*_to_ enhanced by H_2_O_2_**. **(A)** Time course of peak *I*_to_ in a myocyte treated with H_2_O_2_ in the absence and presence of DHA. **(B)** Representative traces of the *I*_to_ under control, in the presence of H_2_O_2_ (200 μM), and H_2_O_2_ + DHA (25 μM), respectively. **(C)** Current–voltage relations of the peak *I*_to_ **(C-a)** and steady state currents (*I*_K,SS_, **C-b**) showing less effects of DHA on enhancement of peak *I*_to_ and *I*_K.ss_ (*n* = 6, *p < 0.05, **p < 0.01 vs. control.). Test potentials ranged from −60 to +50 mV in 10 mV steps. **(D)** Recovery of *I*_to_ from inactivation showing no significant effect of DHA (25 μM) on the *I*_to_ recovery sped-up by H_2_O_2_ (200 μM; *p* > 0.05, *n* = 7).

Consistent with our recent finding (Zhao et al., 2012b), H_2_O_2_ (200 μM) increased the amplitudes of both peak (from 8.94 ± 1.07 to 12.8 ± 1.21 pA/pF at testing potential of 50 mV, *n* = 6, *p* < 0.01) and steady state (late phase at the end of 400 ms pulse; from 2.16 ± 0.25 to 3.13 ± 0.47 pA/pF, *n* = 6, *p* < 0.01) component of *I*_to_. Additionally, H_2_O_2_ also slowed inactivation (τ_s, in_ from 96.6 ± 4.3 to 158.1 ± 5.7 ms; τ_f, in_ from 17.4 ± 1.7 to 24.7 ± 14.0 ms, *n* = 7, *p* < 0.01). However, DHA at 25 μM, the concentration which dramatically suppressed H_2_O_2_-induced EADs, did not show any significant effects on current amplitudes (peak *I*_to_ = 12.51 ± 1.47 pA/pF; *I*_to.ss_ = 3.34 ± 0.31 pA/pF, *n* = 6, *p* > 0.05 compared to H_2_O_2_, respectively) or inactivation process of *I*_to_ (τ_s, in_: 154.6 ± 6.6 ms and τ_f, in_: 23.9 ± 1.1 ms, *n* = 7, *p* > 0.05 compared to H_2_O_2_; Figures 4A–C). Furthermore, we found that H_2_O_2_ accelerated the recovery from inactivation of *I*_to_ mainly by decreasing the fast component (τ_f. re_: from 817.2 ± 79.2 ms to 341.9 ± 26.1 ms, *n* = 7, *p* < 0.05), but not by changing the slow component (τ_s.re_: from control 5335.4 ± 504.8 ms to H_2_O_2_ 4963.2 ± 459.9 ms, *p* > 0.05). Similarly DHA (25 μM) did not cause any significant alteration in *I*_to_ recovery kinetics after H_2_O_2_ treatment (Figure 4D).

### Effect of DHA on intracellular ROS levels

**Figure 5:**
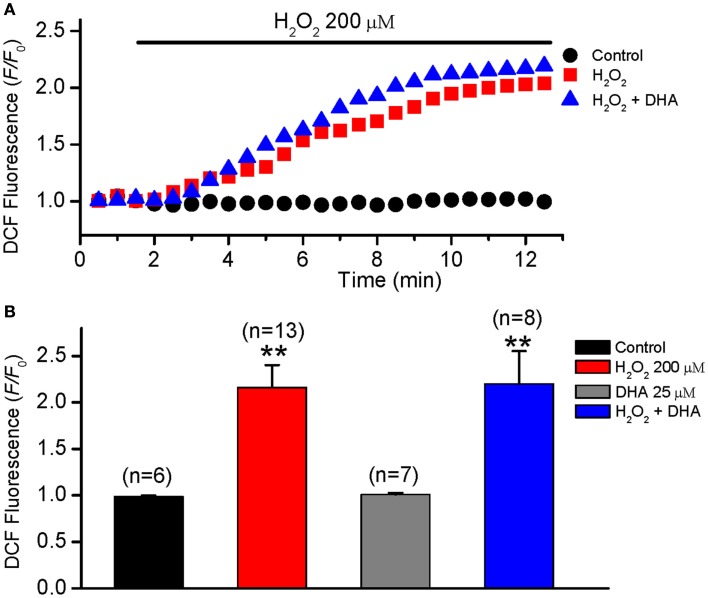
**No effect of DHA on ROS levels in isolated rabbit ventricular myocytes**. ROS levels were measured by monitoring DCF fluorescence intensity in isolated myocytes every 30 s in control, H_2_O_2_ (200 μM) and H_2_O_2_ + DHA (25 μM) groups. **(A)** Time courses of DCF fluorescence intensity (*F/F_0_*) in three representative myocytes from the three groups, respectively. **(B)** Histograms summarizing the DCF intensities for each group measured at 6 min after treatment of H_2_O_2_. **p < 0.01 compared to control. Numbers in parentheses indicate the number of cells in each group.

The level of oxidative stress may either increase or decrease in tissues from humans and animals supplemented with fish oil as reported previously (Garrido et al., 1989; Mas et al., 2010; Tsuduki et al., 2011). To determine whether DHA reduces the incidence of EAD via affecting (decreasing) intracellular ROS, the effect of DHA on intracellular ROS levels was measured in isolated ventricular myocytes treated with exogenous H_2_O_2_ (200 μM) by monitoring CM-DCF fluorescence intensity. The effect of DHA on intracellular ROS levels in the absence of H_2_O_2_ was also measured. As shown in Figure 5, exogenous H_2_O_2_ produced a rapid and dramatic increase in DCF fluorescence intensity in the myocytes and the F/F_0_ of DCF fluorescence intensity reached a steady state value of 2.18 ± 0.24 at 6–10 min after H_2_O_2_ treatment. However, DHA (25 μM, either pretreatment or after treatment) showed no significant effect on the fluorescence of CM-DCF either in the absence or presence of H_2_O_2_ (2.21 ± 0.33, *n* = 8, *p* > 0.05).

## Discussion

Experimental and clinical studies have obtained controversial results regarding the effects of fish oil or ω-3 PUFA on cardiac rhythm (von Schacky, 2008). Differences in the underlying pathogenic mechanisms for the arrhythmia in differing patient groups or animal models may account for these controversies. We and others have previously shown that both exogenous and endogenous ROS-induced EADs can serve as triggers for arrhythmias. In the present study, we provide the first evidence showing that DHA attenuates EADs induced by H_2_O_2_.

The molecular and ionic mechanisms of ion channel modulation by DHA are still not completely understood. A recent review article comprehensively summarized the potential antiarrhythmogenic electrophysiological effect of ω3-PUFAs on the heart (Richardson et al., 2011). Inhibitory effects of DHA on EADs may involve multifactorial mechanisms e.g., (1) via ROS modulation. Although ω3-PUFA may slightly increase levels of oxidative stress due to the susceptibility to oxidation, low to moderate ROS exposure can conversely give rise to up-regulation of antioxidant enzymes and increase antioxidant ability (scavenging ROS) in cardiac tissue (Jahangiri et al., 2006); (2) via direct modulation of ion channels by binding to the channels or affecting cell membrane lipid properties (such as membrane lipid peroxidation). While there is a widespread effect of ω3-PUFA on ion channels and ion pumps, Ca^2+^ and Na^+^ currents are most sensitive to ω3-PUFAs (Richardson et al., 2011). Nevertheless, our present data suggest that the ionic mechanisms underlying inhibitory effect of DHA on EADs most likely involve the direct inhibition on the *I*_Ca,L_ and late *I*_Na_ rather than its putative antioxidant ability. This notion was supported by the observation that there was no effect on CM-DCF fluorescence induced by DHA at the same concentration that led to reduction of EADs. In addition, the fast time course for DHA suppression of *I*_Ca,L_ and late *I*_Na_ also supports a mechanism of direct inhibition of ion channels by DHA. Our most recent data showed H_2_O_2_ also activates *I*_to_ and may facilitate EAD generation (Zhao et al., 2012b). In the present study, however, we showed that DHA did not reverse the *I*_to_ activated by H_2_O_2_ in rabbit ventricular myocytes, which is inconsistent with previous reports that DHA markedly reduces *I*_to_ in human atrial cells and rat ventricular myocytes even at lower concentrations (5–10 μM; Bogdanov et al., 1998; Verkerk et al., 2006; Li et al., 2009). We do not have a ready explanation for this discrepancy, while the molecular subtypes of *I*_to_ proteins might be different between rabbits and other species (including humans) or between different locations in the heart (e.g., ventricle vs. atria). In addition, the H_2_O_2_-activated *I*_to_ seemed to be more resistant to DHA than the *I*_to_ at baseline, since we observed the inhibitory effects of 25 μM DHA on *I*_to_ (up to ∼50%) in the absence of H_2_O_2_.

It has also been reported that n–3-PUFAs are capable of reducing the activity of CaMKII (Zaloga et al., 2006), which may partially account for the inhibitory effect of DHA on EADs. However, since DHA does not alter the ROS level in the presence of H_2_O_2_ (Figure 5), the reduction of CaMKII activity, if any, may be mediated by less Ca entry secondarily to *I*_Ca,L_ blockage, rather than by lower oxidation. Further experiments are needed identify the involvement of CaMKII.

Nevertheless, our present study suggests fish oil supplements may be effective in preventing/treating arrhythmias under an increased oxidative stress condition and serve as an alternative or complimentary anti-arrhythmic drug. Conditions with elevated oxidative stress level including ischemia/reperfusion, heart failure and aging might benefit from fish oil supplements.

## Conflict of Interest Statement

The authors declare that the research was conducted in the absence of any commercial or financial relationships that could be construed as a potential conflict of interest.
